# Novel biomarkers: the RUNX family as prognostic predictors in colorectal cancer

**DOI:** 10.3389/fimmu.2024.1430136

**Published:** 2024-12-09

**Authors:** Yingting Liu, Junjun Chen, An Li, Yue Wu, Junwei Ge, Maoling Yuan, Bin Xu, Xiao Zheng, Lujun Chen, Jingting Jiang

**Affiliations:** ^1^ Department of Tumor Biological Treatment, The Third Affiliated Hospital of Soochow University, Changzhou, Jiangsu, China; ^2^ Jiangsu Engineering Research Center for Tumor Immunotherapy, Changzhou, Jiangsu, China; ^3^ Institute of Cell Therapy, Soochow University, Changzhou, Jiangsu, China

**Keywords:** RUNX, tumor-infiltrating CD8^+^T cells, multi-color immunohistochemical staining, colorectal cancer, prognosis

## Abstract

While biomarkers have been shown to enhance the prognosis of patients with colorectal cancer (CRC) compared to conventional treatments, there is a pressing need to discover novel biomarkers that can assist in assessing the prognostic impact of immunotherapy and in formulating individualized treatment plans. The RUNX family, consisting of RUNX1, RUNX2, and RUNX3, has been recognized as crucial regulators in developmental processes, with dysregulation of these genes also being implicated in tumorigenesis and cancer progression. In our present study, we demonstrated a crucial regulatory role of RUNX in CD8^+^T and CD103^+^CD8^+^T cell-mediated anti-tumor response within the tumor microenvironment (TME) of human CRC. Specifically, RUNXs were significantly differentially expressed between tumor and normal tissues in CRC. Patients with a greater proportion of infiltrating CD8^+^RUNX1^+^, CD103^+^CD8^+^RUNX1^+^, CD8^+^RUNX2^+^, CD103^+^CD8^+^RUNX2^+^, CD8^+^RUNX3^+^, or CD103^+^CD8^+^RUNX3^+^ T cells demonstrated improved outcomes compared to those with lower proportions. Additionally, the proportions of infiltrating CD8^+^RUNX1^+^T and CD8^+^RUNX3^+^T cells may serve as valuable prognostic predictors for CRC patients, independent of other clinicopathological factors. Moreover, further bioinformatic analysis conducted utilizing the TISIDB and TIMER platforms demonstrated significant associations between the members of the RUNX family and immune-infiltrating cells, specifically diverse subpopulations of CD8^+^TILs. Our study of human colorectal cancer tissue microarray (TMA) also revealed positive and statistically significant correlations between the expressions of RUNX1, RUNX2, and RUNX3 in both CD8^+^T cells and CD103^+^CD8^+^T cells. Our study comprehensively revealed the varied expressions and prognostic importance of the RUNX family in human colorectal cancer tissues. It underscored their potential as vital biomarkers for prognostic evaluation in colorectal cancer patients and as promising targets for immunotherapy in treating this disease.

## Introduction

Colorectal cancer (CRC) is a leading cause of morbidity and mortality worldwide. Traditional treatment including surgery, chemotherapy, and radiotherapy could provide benefits for early-stage patients, but side effects and tumor recurrence due to their non-specificity, cytotoxicity, and incompleteness remain significant challenges in treating CRC (1, 2). Immunotherapy is an emerging treatment that leverages patients’ own immune cells to fight tumor cells. It is regarded as a valuable and essential supplementary approach to conventional standard treatments. Certain therapies, such as immune checkpoint inhibitors targeting PD-1/PD-L1 and CAR-T cell therapy, have shown notably effective outcomes in specific tumor types (3, 4). Due to tumor heterogeneity and the immunosuppressive tumor microenvironment, there is still significant potential for enhancing the responsiveness and overall remission rate of immunotherapy in malignant tumors. The current efficacy of immunotherapy in CRC is closely tied to patient-specific biomarkers, such as microsatellite instability, programmed cell death ligands expression and so on. There is a pressing necessity to discover novel biomarkers that can assist in evaluating the prognostic implications of immunotherapy and in devising individualized immunotherapy protocols. Various subtypes and quantities of infiltrating immune cells, particularly tumor-infiltrating T cells (TILs), are valuable indicators for assessing the effectiveness of immunotherapy and predicting patient outcomes (5, 6). Among these subsets, CD8^+^ T cells are deemed essential and preferred for their role in anti-tumor immune responses. Despite their importance, the dysfunction and depletion of these cells can result in tumor immunosuppression and tolerance, allowing tumor growth and progression (7). Addressing these challenges is essential for advancing the role of immunotherapy in the treatment of CRC.

The signature profiles of transcription factors, activating receptors or inhibitory receptors expressed by these tumor-infiltrating CD8^+^T cells play a crucial role in the regulation of their own anti-tumor immune functions. For example, the abundance of intratumorally infiltrating CXCL13^+^CD8^+^T in renal clear cell carcinoma correlates with poor clinical prognosis (8), and the abundance of infiltrating PD-1^+^CD8^+^T cells in gastric carcinoma correlates with improved prognosis (9). In our prior investigation, it was observed that CD226 exhibited elevated expression levels in CD8^+^T cells present within gastric cancer tumor infiltrates. Furthermore, the CD226^+^CD8^+^T cell subset was identified as a significant prognostic indicator for favorable outcomes in gastric patients (10). Therefore, further identification of more effective biomarkers through well-defined CD8^+^ T-cell subpopulations will certainly be beneficial in assisting clinical evaluation of tumor patients.

RUNX, a transcription factor family member, plays a crucial role in regulating mammalian cell proliferation, differentiation, lineage development, osteogenesis, and neurogenesis (11–13). The RUNX gene family comprises three members: RUNX1, RUNX2, and RUNX3. The functionality of RUNX proteins varies depending on the cellular context and is influenced by pathways such as transforming growth factor beta (TGF-β), BMP, WNT, hedgehog, Notch, receptor tyrosine kinase 6, and Yes-associated protein 1 (YAP1), which are associated with major developmental pathways (14–17). Dysregulation of RUNX family gene can lead to a variety of diseases, including tumors (18, 19). Knockout studies show that RUNX1 is crucial for the development and upkeep of the blood system. Runx1-deficient mice die before birth from bleeding in the brain and blood system failure, and RUNX1 is closely linked to leukemia and myelodysplasia in human (20). The fusion oncogene RUNX1/RUNX1T1 could reprogram a large transcriptional network to establish and maintain acute myeloid leukemia (AML) via intricate PPI interactions and kinase-driven phosphorylation events in both adult and pediatric patients (21). RUNX2 plays a critical role in the differentiation of osteoblasts and chondrocytes, and mice lacking functional Runx2 alleles perish shortly after birth as a result of impaired ossification and respiratory failure (22). RUNX2 is thought to be associated with human osteosarcoma progression (23), breast cancer-mediated bone metastasis (24) and has also been reported to related with poor prognosis in cervical (25), bladder (26) and pancreatic (27) cancer. RUNX3 plays an important role in neurogenesis and thymogenesis. It is associated with the development of gastric epithelial cells; the majority of Runx3-deficient mice die of gastric epithelial hyperplasia shortly after birth, and those that survive tend to develop spontaneous inflammatory bowel inflammation (28, 29). The inactivation of RUNX3 in a variety of solid tumors has also been identified as an important tumor oncogenic factor.RUNX3 has been reported to act as a tumor suppressor in melanoma (30), gastric cancer (31) and lung cancer (32), inhibited tumor progression and metastasis. The RUNX family has important regulatory roles in both lineage development and differentiation effects of T cells, but there are fewer studies related to how RUNX family members enhance T lymphocyte function in the tumor microenvironment to treat tumors.

In this study, we aimed to examine the clinical correlations and prognostic significance of RUNX family gene expressions in CD8^+^TILs and CD103^+^CD8^+^TILs in human CRC tissues. We conducted a thorough analysis of RUNX genes across various cancers using TCGA data. Additionally, we explored the regulatory role of RUNX genes in CD8^+^TIL effector function using TIMER and TISIDB databases. We showed that the intensity of CD8^+^RUNX1^+^TILs, CD8^+^RUNX2^+^TILs and CD8^+^RUNX3^+^TILs could be useful prognostic predictors for CRC patients. Additionally, we investigated the interactions between RUNX family genes and immune cells within the colorectal cancer (CRC) tumor microenvironment.

## Materials and methods

### Patients and tissue specimens

The CRC tissue microarray (TMA, HColA180Su21) was provided by Shanghai Outdo Biotech Co. Ltd and contained tumor tissues and corresponding paracancerous tissues from 94 patients. All patients did not receive preoperative neoadjuvant radiotherapy, chemotherapy and immunotherapy, and were operated from February 2012 to September 2014, and were diagnosed with colorectal carcinoma by pathology after surgery. A total of 89 cases were included in this study excluding factors such as core point detachment during the experiment and incomplete clinical follow-up data. Clinical parameters are detailed in **Tables 1**–**4** and the experiment protocol was approved by the ethics committee of the Third Affiliated Hospital of Soochow University.

**Table 1:** Correlation between the infiltrating CD8^+^T and CD103^+^CD8^+^T cells in CRC tissues and clinical parameters of patients.

Clinical parameters		Cases	CD8^+^T				CD103^+^T				CD103^+^CD8^+^T			
			Low	High	χ^2^	*P*	Low	High	χ^2^	*P*	Low	High	χ^2^	*P*
Gender	Male	43	7	36	0.558	0.455	35	8	3.706	0.054	35	8	0.714	0.398
	Female	46	5	41			29	17			34	12		
Age	≤65	45	6	39	0.002	0.967	33	12	0.091	0.763	35	10	0.003	0.954
	>65	44	6	38			31	13			34	10		
Grade	I-II	55	4	51	4.760	***0.029***	40	15	0.048	0.827	42	13	0.112	0.738
	III-IV	34	8	26			24	10			27	7		
AJCC	I-II	54	5	49	2.100	0.147	37	17	0.782	0.377	39	15	2.219	0.136
	III-IV	35	7	28			27	8			30	5		
Tumor size	≤5.3 cm	45	6	39	0.002	0.967	33	12	0.091	0.763	37	8	1.151	0.283
	>5.3 cm	44	6	38			31	13			32	12		
T	T_1-2_	9	1	8	0.048	0.826	6	3	0.136	0.712	6	3	0.678	0.410
	T_3-4_	80	11	69			58	22			63	17		
N	N_0_	56	6	50	0.993	0.319	38	18	1.228	0.268	40	16	3.225	0.073
	N_1-3_	33	6	27			26	7			29	4		
M	M_0_	84	11	73	0.193	0.661	60	24	0.172	0.679	65	19	0.019	0.892
M_1-3_	5	1	4			4	1			4	1		

Values higher than the cutoff point were defined as “High”, and the others were defined as “Low”. Bold italic signifies *P* < 0.05.

**Table 2:** Correlation between the infiltrating RUNX1^+^T, CD8^+^RUNX1^+^T and CD103^+^CD8^+^RUNX1^+^T cells in CRC tissues and clinical parameters of patients.

Clinical parameters		Cases	RUNX1^+^				CD8^+^RUNX1^+^				CD103^+^CD8^+^RUNX1^+^			
			Low	High	χ^2^	*P*	Low	High	χ^2^	*P*	Low	High	χ^2^	*P*
Gender	Male	43	5	38	0.041	0.839	18	25	0.224	0.636	33	10	0.005	0.942
	Female	46	6	40			17	29			35	11		
Age	≤65	45	5	40	0.131	0.717	15	30	1.370	0.242	33	12	0.476	0.490
	>65	44	6	38			20	24			35	9		
Grade	I-II	55	5	50	1.420	0.233	20	35	0.529	0.467	41	14	0.276	0.599
	III-IV	34	6	28			15	19			27	7		
AJCC	I-II	54	6	48	0.198	0.657	19	35	0.987	0.321	38	16	2.773	0.096
	III-IV	35	5	30			16	19			30	5		
Tumor size	≤5.3 cm	45	3	42	2.723	0.099	18	27	0.017	0.895	34	11	0.036	0.849
	>5.3 cm	44	8	36			17	27			34	10		
T	T_1-2_	9	1	8	0.014	0.904	4	5	0.110	0.740	6	3	0.527	0.468
	T_3-4_	80	10	70			31	49			62	18		
N	N_0_	56	6	50	0.377	0.539	20	36	0.826	0.364	39	17	3.830	0.050
	N_1-3_	33	5	28			15	18			29	4		
M	M_0_	84	10	74	0.286	0.593	33	51	0.001	0.975	64	20	0.038	0.845
	M_1-3_	5	1	4			2	3			4	1		

Values higher than the cutoff point were defined as “High”, and the others were defined as “Low”.

**Table 3:** Correlation between the infiltrating RUNX2^+^T, CD8^+^RUNX2^+^T and CD103^+^CD8^+^RUNX2^+^T cells in CRC tissues and clinical parameters of patients.

Clinical parameters		Cases	RUNX2+				CD8+RUNX2+				CD103+CD8+RUNX2+			
			Low	High	χ^2^	*P*	Low	High	χ^2^	*P*	Low	High	χ^2^	*P*
Gender	Male	43	25	18	3.259	0.071	36	7	1.831	0.176	34	9	0.328	0.567
	Female	46	35	11			33	13			34	12		
Age	≤65	45	27	18	2.279	0.131	34	11	0.203	0.652	34	11	0.036	0.849
	>65	44	33	11			35	9			34	10		
Grade	I-II	55	39	16	0.800	0.371	42	13	0.112	0.738	43	12	0.252	0.615
	III-IV	34	21	13			27	7			25	9		
AJCC	I-II	54	34	20	1.239	0.266	38	16	4.038	***0.044***	37	17	4.737	***0.030***
	III-IV	35	26	9			31	4			31	4		
Tumor size	≤5.3 cm	45	31	14	0.090	0.764	36	9	0.319	0.572	37	8	1.709	0.191
	>5.3 cm	44	29	15			33	11			31	13		
T	T_1-2_	9	5	4	0.641	0.423	6	3	0.678	0.410	7	2	0.010	0.918
	T_3-4_	80	55	25			63	17			61	19		
N	N_0_	56	35	21	1.661	0.197	39	17	5.390	***0.020***	39	17	3.830	0.050
	N_1-3_	33	25	8			30	3			29	4		
M	M_0_	84	58	26	1.813	0.178	66	18	0.934	0.334	63	21	1.636	0.201
	M_1-3_	5	2	3			3	2			5	0	

Values higher than the cutoff point were defined as “High”, and the others were defined as “Low”. Bold italic signifies *P* < 0.05.

**Table 4:** Correlation between the infiltrating RUNX3^+^T, CD8^+^RUNX3^+^T and CD103^+^CD8^+^RUNX3^+^T cells in CRC tissues and clinical parameters of patients.

Clinical parameters		Cases	RUNX3^+^				CD8^+^RUNX3^+^				CD103^+^CD8^+^RUNX3^+^			
			Low	High	χ^2^	*P*	Low	High	χ^2^	*P*	Low	High	χ^2^	*P*
Gender	Male	43	29	14	0.729	0.393	6	37	0.616	0.433	33	10	0.180	0.671
	Female	46	27	19			4	42			37	9		
Age	≤65	45	28	17	0.019	0.890	5	40	0.001	0.970	33	12	1.533	0.216
	>65	44	28	16			5	39			37	7		
Grade	I-II	55	38	17	2.349	0.125	4	51	2.267	0.132	46	9	2.130	0.144
	III-IV	34	18	16			6	28			24	10		
AJCC	I-II	54	34	20	0.000	0.992	4	50	2.018	0.155	41	13	0.608	0.436
	III-IV	35	22	13			6	29			29	6		
Tumor size	≤5.3 cm	45	29	16	0.091	0.764	5	40	0.001	0.970	37	8	0.691	0.406
	>5.3 cm	44	27	17			5	39			33	11		
T	T_1-2_	9	5	4	0.233	0.629	0	9	1.267	0.260	7	2	0.005	0.946
	T_3-4_	80	51	29			10	70			63	17		
N	N_0_	56	35	21	0.011	0.915	5	51	0.806	0.369	43	13	0.313	0.576
	N_1-3_	33	21	12			5	28			27	6		
M	M_0_	84	54	30	1.193	0.275	9	75	0.408	0.523	67	17	1.098	0.295
	M_1-3_	5	2	3			1	4			3	2	

Values higher than the cutoff point were defined as “High”, and the others were defined as “Low”.

### Multi-color immunohistochemically staining

The multi-color immunohistochemically staining (mIHC) was performed using the Opal 5-color fluorescent IHC kit (catalog No. NEL811001KT, PerkinElmer, USA) and automated quantitative analysis (PerkinElmer, USA) to detect CD8, CD103, RUNX1, RUNX2, and RUNX3 in tumor tissues. The 4’, 6-diamidino-2-phenylindole (DAPI) was utilized for nuclear staining. Subsequently, the concentrations of the six antibodies listed above were individually optimized against the markers, and a spectral library was established using single-stained slides. The PC TMA slide underwent dewaxing and rehydration via a sequence of xylene-to-alcohol washes prior to immersion in distilled water. Heat-induced antigen retrieval was done in citric acid solution (PH=6.0), followed by mIHC staining with primary antibodies including anti-CD8A (1:200, CST70306, Cell Signaling Technology), anti-CD103 (1:1000, ab224202, Abcam), anti-RUNX1 (1:1000, ab240639, Abcam), anti-RUNX2 (1:1000, ab192256, Abcam), and anti-RUNX3 (1:500, ab40278, Abcam). The PC TMA slide was then incubated with HRP-conjugated secondary antibodies in Opal working solution (PerkinElmer, USA) and mounted with ProLong Diamond Antifade Reagent with DAPI (Thermofisher, USA).

### Imaging analysis

The TissueFAXS system, developed by TissueGnostics Asia Pacific Limited in Austria, was utilized for panoramic multispectral scanning of PC TMA slides. The resulting images were analyzed using StrataQuest software (Version No. 7.0.1.165, TissueGnostics Asia Pacific Limited, Austria), wherein each fluorophore was spectrally unmixed into distinct channels and saved as individual files. DAPI was employed to create a binary mask representing all viable cells within the image. The expressions of RUNX1, RUNX2, and RUNX3 were utilized in conjunction with DAPI to generate binary masks representing cells expressing these biomarkers. Furthermore, binary masks of CD8 and CD103 were employed to assess the infiltration intensity of lymphocytes. Each slide was reviewed independently by two senior pathologists who were unaware of the patients’ clinical characteristics. The immunohistochemical staining was evaluated utilizing the H-score methodology, consistent with our prior publications (33, 34).

### Databases applied to analyze RUNX expression in human tumor and immune estimation

The gene expression analyses of the RUNX family in various cancer types were conducted utilizing the publicly available web resources GEPIA (http://gepia.cancer-pku.cn/) and UALCAN (http://ualcan.path.uab.edu/), which analyze cancer transcriptome data from TCGA and MET500 (35). Additionally, the web-based interactive platform TIMER2.0 (https://cistrome.shinyapps.io/timer/) was utilized to systematically analyze immune infiltration in different malignancies (36, 37). The TIMER2.0 database utilizes six sophisticated algorithms to conduct a comprehensive assessment of tumor-infiltrating lymphocyte (TIL) levels in The Cancer Genome Atlas (TCGA) or other tumor-related datasets. Furthermore, the database is capable of accurately estimating tumor purity. Our study examined the expression of RUNXs in different types of cancers and explored the correlation between RUNXs expression and TILs using gene modules. Moreover, an examination of the correlation between the expression of RUNXs and gene markers associated with tumor-infiltrating lymphocytes (TILs), such as CD8^+^/CD4^+^ T cells, B cells, monocytes, natural killer (NK) cells, dendritic cells (DCs), tumor-associated macrophages (TAMs), M1 macrophages, M2 macrophages, neutrophils, T cells, and their respective subtypes, has been conducted using correlation modules. The TISIDB database, accessible at http://cis.hku.hk/TISIDB/index.php, serves as an online integrated repository portal that aggregates numerous human cancer datasets from the TCGA database. In the TISIDB database, RUNX gene expression was correlated with immune subtypes or molecular subtypes of different cancer types. Differences with a *P* value 0.05 were considered statistically significant.

### Statistical analyses

Statistical analyses were conducted using Prism 9 software and RStudio 6.3 to compare disease-related factors in patients with varying levels of RUNX1, RUNX2, and RUNX3 expression. The fluorescence intensity of each protein between different patients was truncated using the surv_cutpoint function in the survminer package of the R software package, and the patients were divided into two groups of high and low expression according to the cutoff point truncation value. The survival curve was plotted using the R package survival. Cox model analysis was conducted using the coxph function for both uni-variate and multi-variate analysis. Log-rank survival analysis was used to predict postoperative overall survival (OS) with significance set at *P* < 0.05.

## Result

### RUNXs are significantly differentially expressed between tumor and normal tissues in various human cancers

**Figure 1:**
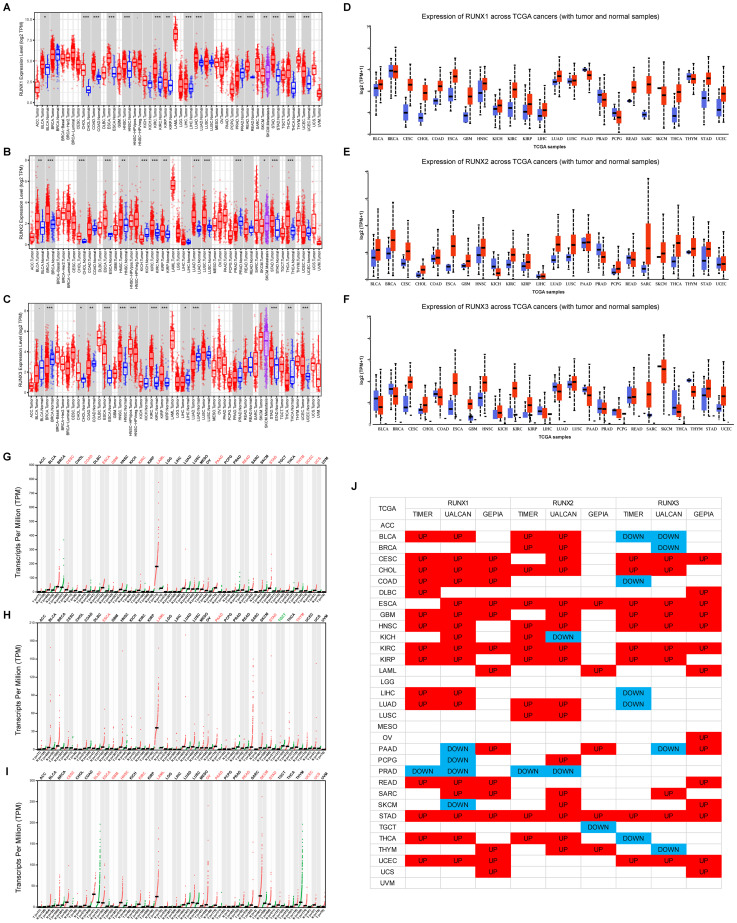
A comparison of expression levels of RUNX genes in human cancers of different types. **(A–C)** RUNX1, RUNX2 and RUNX3 gene levels in different cancer types (red) and normal tissue (blue) available in TIMER database. **(D–F)** RUNX1, RUNX2 and RUNX3 gene levels in different cancer types (red) and normal tissue (blue) available in UALCAN database. **(G–I)** RUNX1, RUNX2 and RUNX3 gene levels in different cancer types (red) and normal tissue (blue) available in GEPIA database. **(J)** Pan-cancer landscape of differential expression of RUNX1, RUNX2, and RUNX3 across three different TCGA databases. *P< 0.05, **P< 0.01, and ***P< 0.001.

The RUNX gene family exhibited abnormal expression patterns in various human cancers. Analysis of TCGA data through TIMER, UALCAN, and GEPIA databases showed distinct expression profiles for RUNX family members across different tumor types, generally indicating upregulation in most tumors (**Figure 1**). RUNX1 and RUNX2 had similar cancer expression profiles, with overexpression in most cancer types and downregulation in prostate adenocarcinoma (PRAD). In contrast, RUNX3 was more frequently downregulated in several cancer types, including BLCA, COAD, LIHC, LUAD, THCA, and THYM. Notably, RUNX1 was upregulated, while RUNX3 was downregulated in COAD tumor tissues, with statistical significance. However, RUNX2 showed no significant difference in expression between tumor and normal tissues in COAD (refer to **Figures 1A–C**). These results suggest a progressive downregulation trend of RUNX1, RUNX2, and RUNX3 in COAD tumor tissues, indicating a potential role for RUNX3 and RUNX2 as tumor suppressors in COAD, in contrast to RUNX1.

### Expressions and localization of CD8, CD103 and RUNX family genes in CRC tissues and normal colorectal tissues

**Figure 2:**
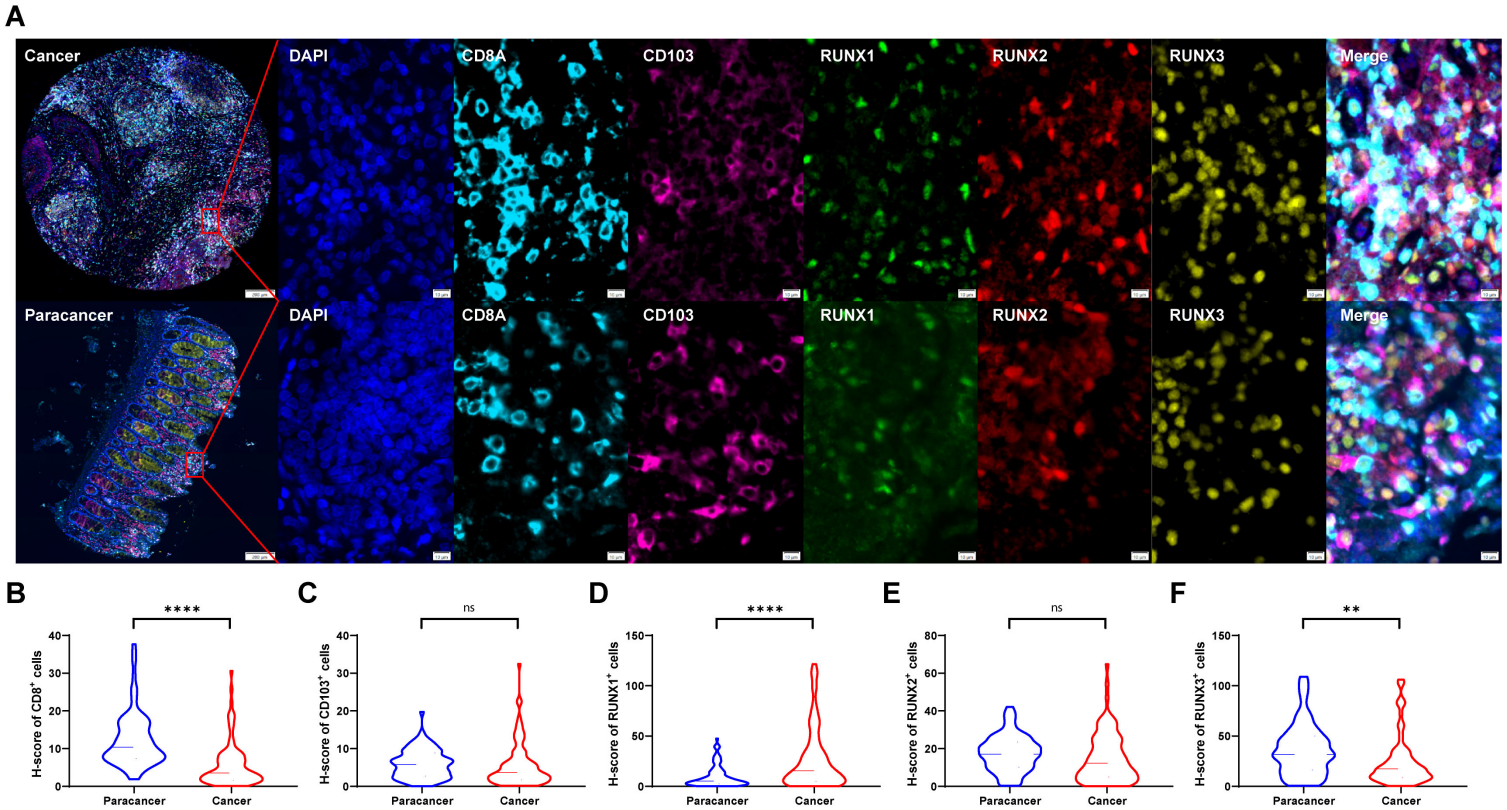
The expressions of CD8, CD103, RUNX1, RUNX2, and RUNX3 in human CRC TMA. **(A)** Multi-spectral immunohistochemistry (mIHC) was utilized to obtain images from human colorectal cancer tissue and adjacent paracancer tissue. **(B–F)** H-scores of CD8^+^ **(B)**, CD103^+^ **(C)**, RUNX1^+^ **(D)**, RUNX2^+^ **(E)**, and RUNX3^+^ **(F)** cells. **P < 0.01, ****P < 0.0001 and ns for no significance.

In our previous study, we have already found that CD8^+^T and tissue-resident CD103^+^CD8^+^T cells were prognostic factors for colorectal cancer (38). In this study, we further explored the expression levels of RUNX family members on these two types of tumor-infiltrating lymphoid cells. We examined the localization of CD8, CD103, RUNX1, RUNX2 and RUNX3 in the CRC TMA slide by mIHC. **Figure 2A** showed that the RUNX1 (green), RUNX2 (red) and RUNX3 (yellow) could be predominantly detected in the nucleus while CD8 (blue) and CD103 (pink) were expressed on the membrane and cytoplasm. Based on the H-score analysis, CRC cancer tissues exhibited a significantly elevated presence of CD8^+^ cells in comparison to adjacent paracancerous tissues (**Figure 2B**). Furthermore, the expression levels of RUNX1 were notably higher in cancerous tissues as opposed to paracancerous tissues (**Figure 2D**), whereas the expression of RUNX3 was comparatively lower in cancerous tissues relative to paracancerous tissues (**Figure 2F**). Conversely, there was no discernible distinction in the levels of CD103^+^ cells and RUNX2 expression between cancerous and paracancerous tissues (**Figures 2B, E**, respectively). These results of RUNX family members between cancer and paracancer were consistent with the previous results in the TCGA database (**Figures 1A–C**).

**Figure 3:**
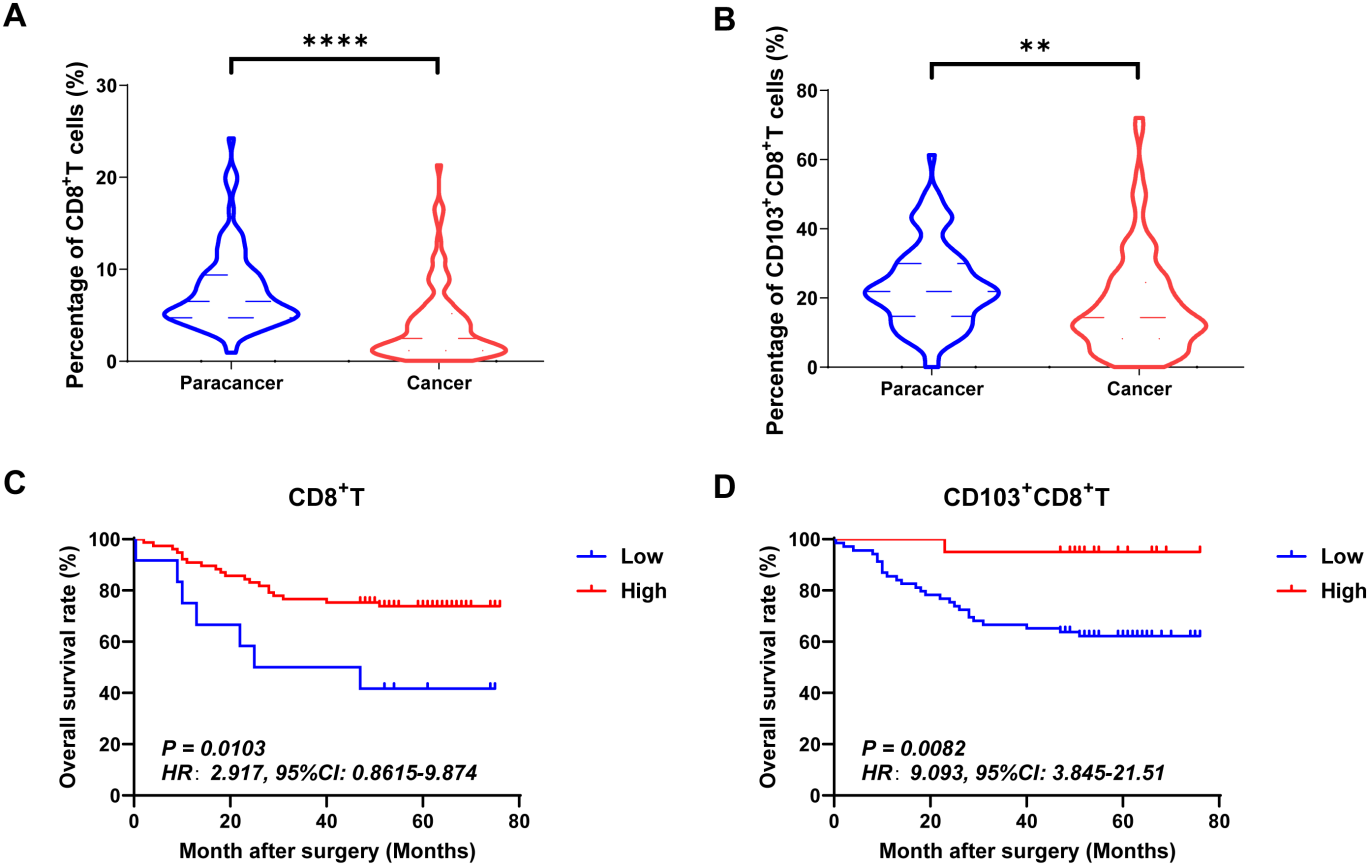
The expressions and prognostic values of CD8^+^TILs and CD103^+^CD8^+^TILs in human CRC TMA. **(A, B)** The populations of CD8^+^TILs and CD103^+^CD8^+^TILs were compared between CRC tissue and paracancer tissue. **(C, D)** The prognostic value of CD8^+^TILs and CD103^+^CD8^+^TILs were compared between CRC tissue and paracancer tissue. **P < 0.01, ****P < 0.0001.

Tumor-infiltrating cytotoxic CD8^+^ T cells effectively inhibited tumor growth. Based on mIHC and imaging analysis (**Figure 2A**), membrane staining of CD103 could be found on infiltrating immune cells. The tissues-resident CD8^+^T cells, which were defined as CD103^+^CD8^+^T cells, were found in both adjacent normal and tumor tissues. In the present study, we also found that the proportions of CD8^+^ T cells and CD103^+^CD8^+^ T cells were significantly higher in human CRC tissues than in paraneoplastic tissues (**Figures 3A, B**). Herein, we also investigated the relationship between the frequency of tumor-infiltrating CD8^+^T and CD103^+^CD8^+^T cells and CRC patients’ survival. **Figures 3C, D** showed that patients with high frequency of CD8^+^ TILs (HR=2.917, 95% CI: 0.8615-9.874, *P*=0.0103, **Figure 3C**) and CD103^+^CD8^+^ TILs (HR=9.093, 95% CI: 3.845-21.51, *P*=0.0082, **Figure 3D**) had a better prognosis.

### Prognostic value of RUNX1, RUNX2 and RUNX3 expression in CD8^+^TILs and CD103^+^CD8^+^TILs in human CRC

**Figure 4:**
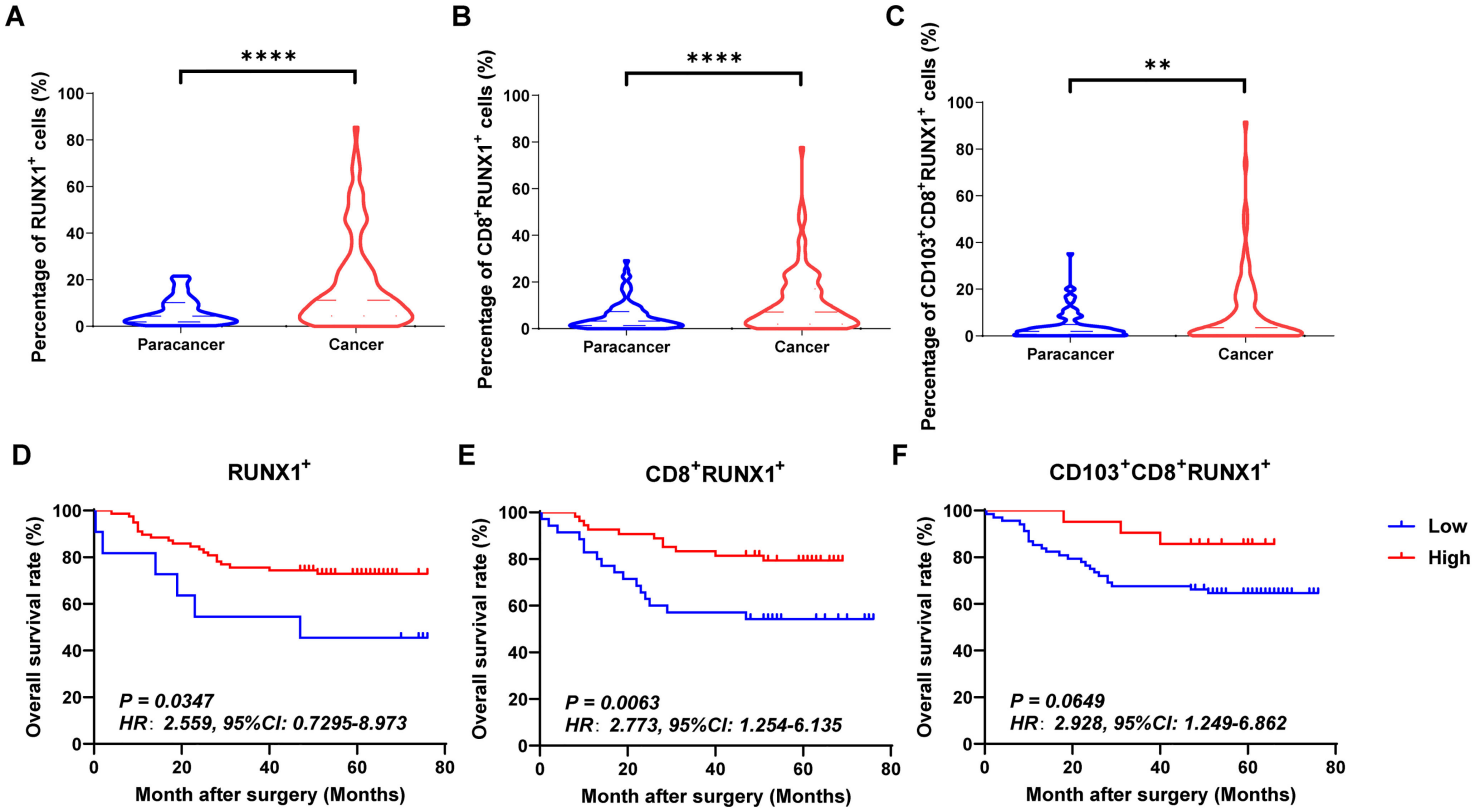
The expressions and prognostic values of RUNX1^+^TILs, CD8^+^RUNX1^+^TILs and CD103^+^CD8^+^RUNX1^+^TILs in human CRC TMA. **(A–C)** The populations of RUNX1^+^TILs, CD8^+^RUNX1^+^TILs and CD103^+^CD8^+^RUNX1^+^TILs were compared between CRC tissue and paracancer tissue. **(D–F)** The prognostic significance of RUNX1^+^TILs, CD8^+^RUNX1^+^TILs and CD103^+^CD8^+^RUNX1^+^TILs were assessed between CRC tissue and paracancer tissue. **P < 0.01, ****P < 0.0001.

**Figure 5:**
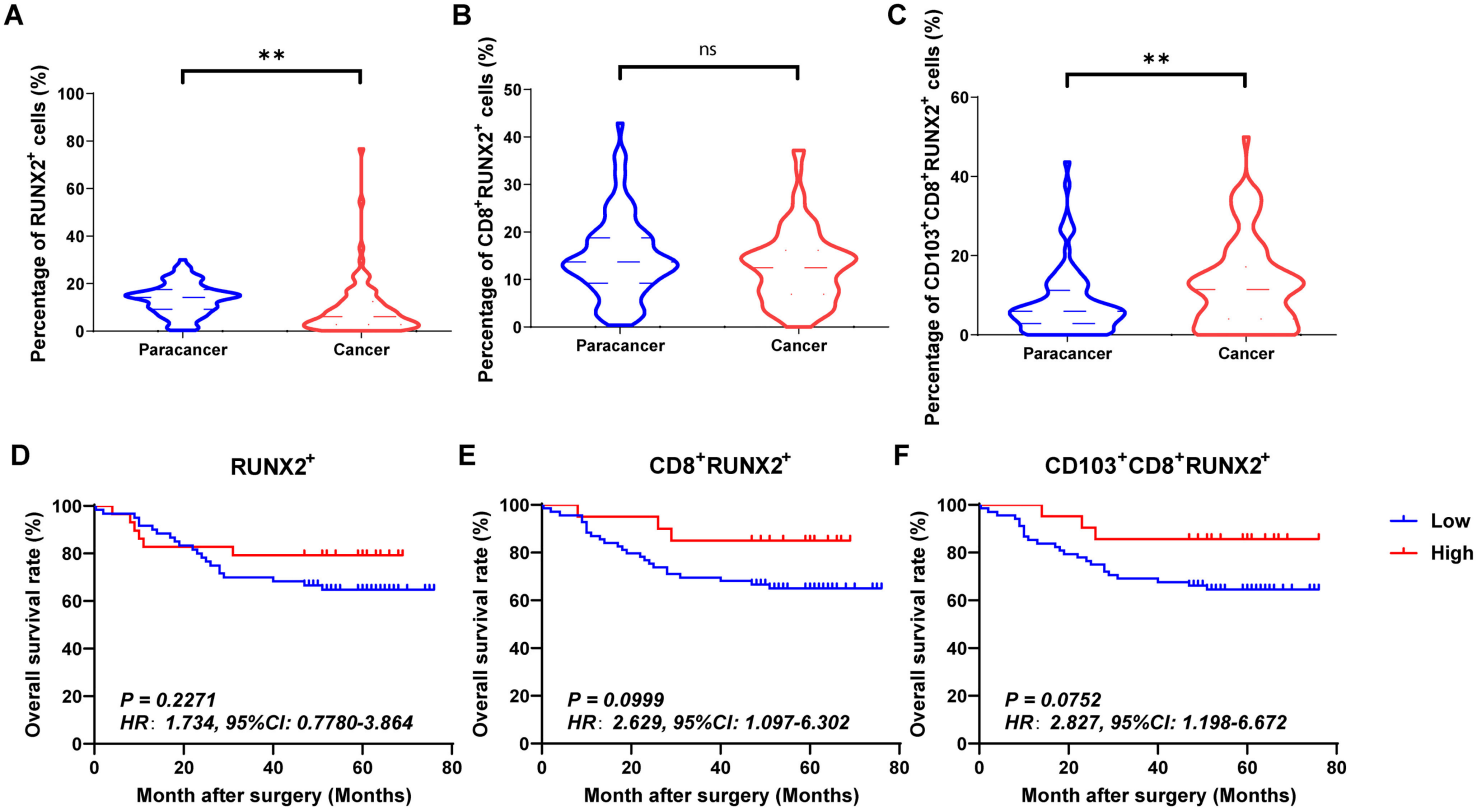
The expressions and prognostic values of RUNX2^+^TILs, CD8^+^RUNX2^+^TILs and CD103^+^CD8^+^RUNX2^+^TILs in human CRC TMA. **(A–C)** The populations of RUNX2^+^TILs, CD8^+^RUNX2^+^TILs and CD103^+^CD8^+^RUNX2^+^TILs were compared between CRC tissue and paracancer tissue. **(D–F)** The prognostic value of RUNX2^+^TILs, CD8^+^RUNX2^+^TILs and CD103^+^CD8^+^RUNX2^+^TILs were compared between CRC tissue and paracancer tissue. **P < 0.01, and ns for no significance.

**Figure 6:**
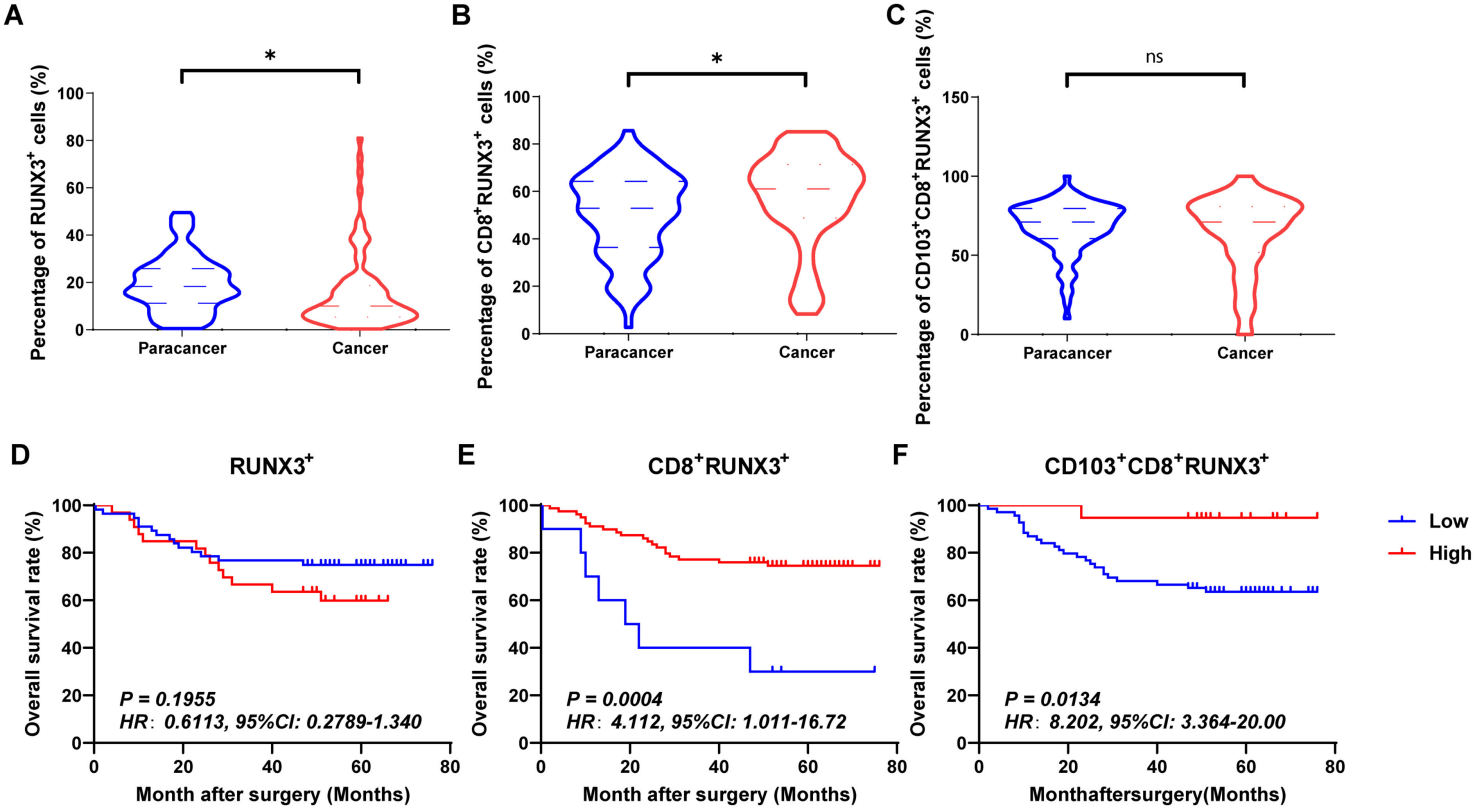
The expressions and prognostic values of RUNX3^+^TILs, CD8^+^RUNX3^+^TILs and CD103^+^CD8^+^RUNX3^+^TILs in human CRC TMA. **(A–C)** The populations of RUNX3^+^TILs, CD8^+^RUNX3^+^TILs and CD103^+^CD8^+^RUNX3^+^TILs were compared between CRC tissue and paracancer tissue. **(D–F)** The prognostic value of RUNX3^+^TILs, CD8^+^RUNX3^+^TILs and CD103^+^CD8^+^RUNX3^+^TILs were compared between CRC tissue and paracancer tissue. *P < 0.05, and ns for no significance.

**Figures 4A–C**, **5A–C** and **6A–C** showed the percentages of RUNX1^+^ cells, CD8^+^RUNX1^+^T/CD8^+^T cells, CD103^+^CD8^+^RUNX1^+^T/CD8^+^T cells, RUNX2^+^ cells, CD8^+^RUNX2^+^T/CD8^+^T cells, CD103^+^CD8^+^RUNX2^+^T/CD8^+^T cells, RUNX3^+^ cells, CD8^+^RUNX3^+^T/CD8^+^T cells and CD103^+^CD8^+^RUNX3^+^T/CD8^+^T cells comparison between paracancer tissues and cancer tissues in CRC. The percentages of RUNX1^+^ cells, CD8^+^RUNX1^+^T/CD8^+^T cells, and CD103^+^CD8^+^RUNX1^+^T/CD8^+^T cells were all higher in cancer tissues than in paracancer tissues (**Figures 4A–C**). The percentage of RUNX2^+^ cells was lower in cancer tissues than in paracancer tissues (**Figure 5A**), while the percentage of CD103^+^CD8^+^RUNX2^+^T/CD8^+^T cells was higher in cancer tissues than in paracancer tissues (**Figure 5C**). These two differences were both statistically significant. Interestingly, the difference in the percentage of CD8^+^RUNX2^+^T among CD8^+^T cells between cancer or paracancer tissues was not statistically significant (**Figure 5B**). We also observed that patients with higher density of CD8^+^RUNX2^+^T cells or CD103^+^CD8^+^RUNX2^+^T cells proportion tend to have a better OS than lower density (HR=2.629, 95% CI: 1.097-6.302, *P*=0.0999, **Figure 5B**; HR=2.827, 95% CI: 1.198-6.672, *P*=0.0752, **Figure 5C**). The percentages of RUNX3^+^ cells (**Figure 6A**) and CD8^+^RUNX3^+^T/CD8^+^T cells (**Figure 6B**) were all lower in cancer tissues than in paracancer tissues. These two differences were both statistically significant, while the difference in the percentage of CD103^+^CD8^+^RUNX3^+^T among CD8^+^T cells between cancer or paracancer tissues was not statistically significant (**Figure 6C**).

Interestingly, we observed that patients with a higher density of CD8^+^RUNX1^+^T cells, CD103^+^CD8^+^RUNX1^+^T cells, CD8^+^RUNX2^+^T cells, CD103^+^CD8^+^RUNX2^+^T cells, CD8^+^RUNX3^+^T cells, or CD103^+^CD8^+^RUNX3^+^T cells proportion all presented a better OS than those with lower density (HR=2.773, 95% CI: 1.254-6.135, *P*=0.0063, **Figure 4E**; HR=2.928, 95% CI: 1.249-6.862, *P*=0.0649, **Figure 4F**; HR=2.629, 95% CI: 1.097-6.302, *P*=0.0999, **Figure 5E**; HR=2.827, 95% CI: 1.198-6.672, *P*=0.0752, **Figure 5F**; HR=4.112, 95% CI: 1.011-16.72, *P*=0.0134, **Figure 6E**; HR=8.202, 95% CI: 3.364-20.00, *P*=0.0134, **Figure 6F**).

### Correlations between patients’ clinical parameters and the intensities of tumor-infiltrating CD8^+^T cells, CD103^+^CD8^+^T cells, CD8^+^RUNXs^+^T cells and CD103^+^CD8^+^RUNXs^+^T cells in human CRC tissues

In the current investigation, we sought to examine the associations between clinical parameters of patients and the levels of tumor-infiltrating CD8^+^T cells, CD103^+^CD8^+^T cells, CD8^+^RUNXs^+^T cells and CD103^+^CD8^+^RUNXs^+^T cells in human CRC tissues. **Table 1** showed the frequency of CD8^+^T cells was positively associated with tumor grade (*P*=0.029, **Table 1**). Additionally, **Table 3** demonstrated a significant association between the frequencies of CD8^+^RUNX2^+^TILs and CD103^+^CD8^+^RUNX2^+^TILs with AJCC stage (*P*=0.044, *P*=0.030, respectively, **Table 3**), as well as a negative and significant association between the frequency of CD8^+^RUNX2^+^TILs and N stage (*P*=0.020, **Table 3**). However, based on the data presented in **Tables 2**, **4**, there was no significant correlation observed between the frequencies of various cell types (RUNX1^+^, CD8^+^RUNX1^+^T, CD103^+^CD8^+^RUNX1^+^T, RUNX3^+^, CD8^+^RUNX3^+^T, and CD103^+^CD8^+^RUNX3^+^T cells) and clinical parameters in colorectal cancer tissues.

Moreover, **Table 5** revealed that individuals with a pathological grade of III+IV exhibit a markedly higher risk of mortality (multi-variate: HR=2.416, 95% CI: 1.085-5.398, *P*=0.031) in comparison to those with a pathological grade of I+II, even after adjusting for variables such as gender, age, tumor size, infiltration of CD8^+^T, infiltration of CD103^+^CD8^+^T, and other relevant factors. Patients with an AJCC stage of T3+T4 demonstrated a significantly elevated risk of death (multi-variate: HR=3.566, 95% CI:1.558-8.160, *P*=0.003) when compared to patients with an AJCC stage of T1+T2. **Table 5** illustrated that patients exhibiting elevated levels of infiltrating CD8^+^T, CD103^+^CD8^+^T, RUNX1^+^T, CD8^+^RUNX1^+^T, CD8^+^RUNX2^+^T, CD8^+^RUNX3^+^T and CD103^+^CD8^+^RUNX3^+^T cells demonstrated a reduced risk of mortality in comparison to patients with lower levels (uni-variate: HR=0.342, 95% CI: 0.144-0.809, *P*=0.015; HR=0.348, 95% CI: 0.162-0.744, *P*=0.006; HR=0.390, 95% CI: 0.157-0.968, *P*=0.0.42; HR=0.359, 95% CI: 0.166-0.775, *P*=0.009; HR=0.467, 95% CI: 0.168-1.297, *P*=0.031; HR=0.240, 95% CI: 0.101-0.571, *P*=0.001; HR=0.362, 95% CI: 0.158-0.828, *P*=0.016, respectively). Besides, the percentages of infiltrating CD103^+^CD8^+^T cells, CD8^+^RUNX1^+^T cells and CD8^+^RUNX3^+^T cells could also serve as significant prognostic indicators for predicting the survival outcomes of CRC patients, irrespective of other clinicopathological variables including gender, age, tumor size, pathological grade, and AJCC stage (**Table 5**).

**Table 5:** Cox model analysis for the correlation between the expression of RUNX, infiltrating CD8^+^T, CD103^+^CD8^+^T cells and clinical parameters of patients.

Clinical parameters	Uni-variate		Multi-variate	
	HR (95% Cl)	*P*	HR (95% Cl)	*P*
Gender (M/F)	1.141 (0.534-2.439)	0.733	0.724 (0.321-1.629)	0.435
Age (years) (>65/≤65)	0.782 (0.366-1.670)	0.525	0.790 (0.363-1.719)	0.552
Tumor size (cm) (>5.3 cm/≤5.3 cm)	0.754 (0.353-1.612)	0.467	0.732 (0.333-1.608)	0.437
Pathological grade (III+IV/I+II)	2.497 (1.167-5.341)	***0.018***	2.416 (1.085-5.398)	***0.031***
AJCC stage (T3+T4/T1+T2)	4.039 (1.809-9.021)	***0.001***	3.566 (1.558-8.160)	***0.003***
Percentage of infiltrating CD8^+^T cells (high/low)	0.342 (0.144-0.809)	***0.015***	1.938 (0.770-4.877)	0.160
Percentage of infiltrating CD103^+^CD8^+^T cells (high/low)	0.348 (0.162-0.744)	***0.006***	2.429 (1.106-5.338)	***0.027***
Percentage of infiltrating RUNX1^+^T cells (high/low)	0.390 (0.157-0.968)	***0.042***	3.111 (1.184-8.176)	***0.021***
Percentage of infiltrating CD8^+^RUNX1^+^T cells (high/low)	0.359 (0.166-0.775)	***0.009***	2.801 (1.253-6.260)	***0.012***
Percentage of infiltrating CD103^+^CD8^+^RUNX1^+^T cells (high/low)	0.341 (0.103-1.133)	0.079	2.284 (0.662-7.881)	0.191
Percentage of infiltrating RUNX2^+^T cells (high/low)	0.576 (0.232-1.429)	0.234	1.761 (0.687-4.510)	0.238
Percentage of infiltrating CD8^+^RUNX2^+^T cells (high/low)	0.467 (0.168-1.297)	***0.031***	2.724 (1.098-6.758)	0.144
Percentage of infiltrating CD103^+^CD8^+^RUNX2^+^T cells (high/low)	0.354 (0.106-1.174)	0.090	1.969 (0.565-6.862)	0.288
Percentage of infiltrating RUNX3^+^T cells (high/low)	1.637 (0.769-3.484)	0.201	0.665 (0.311-1.423)	0.293
Percentage of infiltrating CD8^+^RUNX3^+^T cells (high/low)	0.240 (0.101-0.571)	***0.001***	2.884 (1.161-7.161)	***0.022***
Percentage of infiltrating CD103^+^CD8^+^RUNX3^+^T cells (high/low)	0.362 (0.158-0.828)	***0.016***	2.272 (0.932-5.535)	0.071

Bold italic signifies *P* < 0.05.

### Correlation between the expression levels of RUNX1, RUNX2, RUNX3 and immune infiltration in colorectal cancer

**Figure 7:**
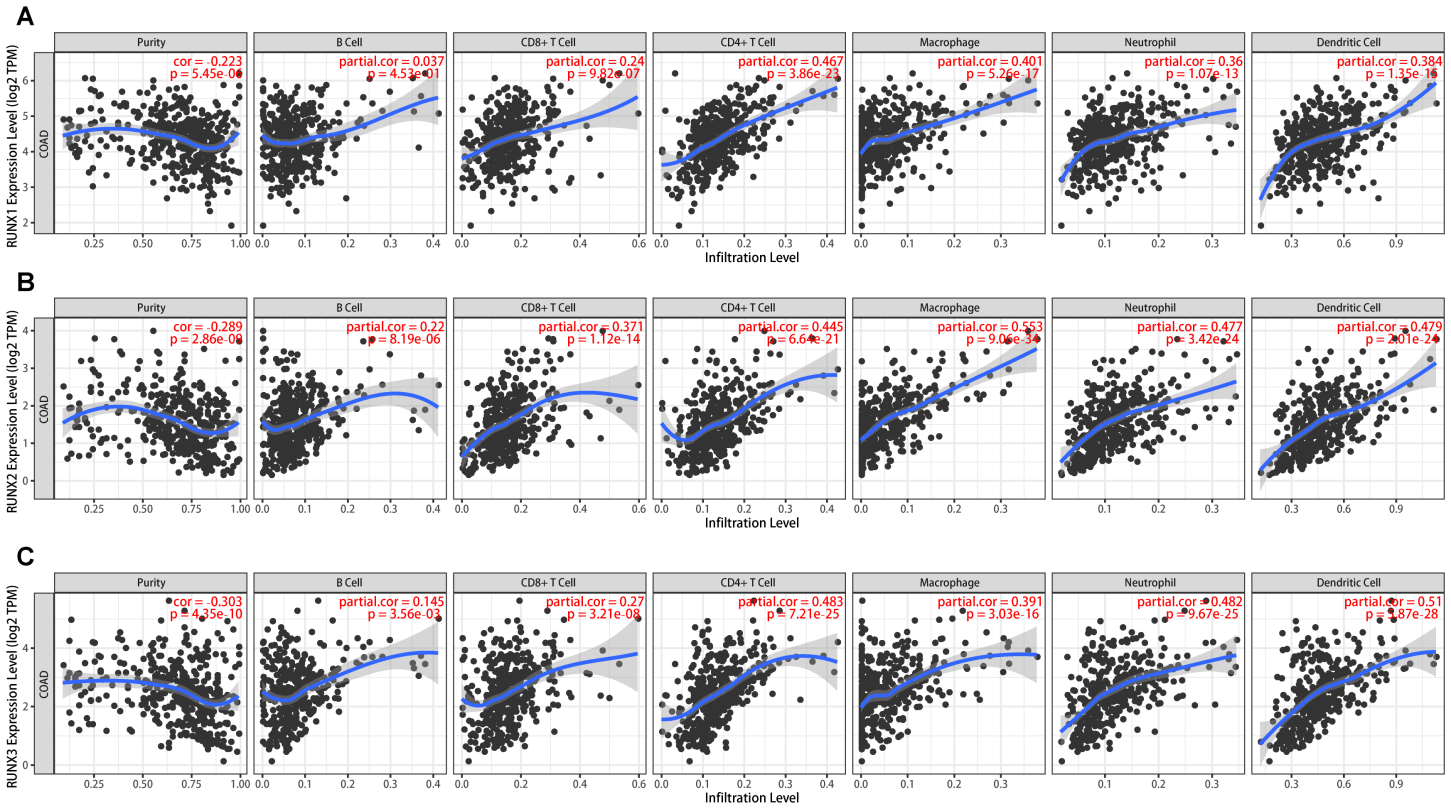
The association between RUNX family gene and cell subsets in human colorectal cancer based on TIMER platform. **(A)** The scatter plots showed the association between RUNX1 expression and B cell, CD8^+^T, CD4^+^T, macrophage, neutrophil, dendritic cell and tumor purity. **(B)** The scatter plots showed the association between RUNX2 expression and B cell, CD8^+^T, CD4^+^T, macrophage, neutrophil, dendritic cell and tumor purity. **(C)** The scatter plots showed the association between RUNX3 expression and B cell, CD8^+^T, CD4^+^T, macrophage, neutrophil, dendritic cell and tumor purity.

Immune infiltration plays a vital role in tumor progression. The TIMER and TISIDB platforms were utilized to examine the association between RUNX family genes and immune cell infiltration in COAD. **Figure 7** showed a significant correlation between the expression of RUNX1, RUNX2, and RUNX3 and the abundance of TILs in COAD, according to the TIMER database. For instance, RUNX1 expression was positively correlated with infiltrating degree of CD8^+^T cell (rho=0.24), CD4^+^T cell (rho=0.467), B cell (rho=0.037), macrophage (rho=1.401), neutrophil (rho=0.36) and dendritic cell (rho=0.384) (**Figure 7A**). RUNX2 expression was positively correlated with infiltrating degree of CD8^+^T cell (rho=0.371), CD4^+^T cell (rho=0.445), B cell (rho=0.22), macrophage (rho=0.553), neutrophil (rho=0.477) and dendritic cell (rho=0.479) (**Figure 7B**). RUNX3 expression was positively correlated with infiltrating degree of CD8^+^T cell (rho=0.27), CD4^+^T cell (rho=0.483), B cell (rho=0.145), macrophage (rho=0.391), neutrophil (rho=0.482) and dendritic cell (rho=0.51) (**Figure 7C**). All the *P*-values were significantly less than 0.001. Furthermore, an examination was conducted on the correlation between RUNX family genes and various biomarkers of TILs (including CD8+ T cells, CD4+ T cells, NK cells, B cells, monocytes, DCs, TAMs, M1/M2 macrophages, neutrophils, T cells, and related subtypes) in COAD using data from the GEPIA database (refer to **Table 6**). The findings suggest that the RUNX1, RUNX2, and RUNX3 genes are correlated with a significant portion of tumor-infiltrating lymphocyte markers in colorectal adenocarcinoma. Various subsets of T cells, such as Th1, Th2, Tfh, Treg, resident, cytotoxic, exhausted, and effector memory T cells, were also analyzed. The RUNX family genes are implicated in the regulation of immune infiltration in colorectal cancer, as shown in **Table 6**.

**Table 6:** Correlation analysis between RUNX family genes and related gene markers of monocyte, macrophages, and T-cell exhaustion in Gene Expression Profiling Interaction Analysis (GEPIA).

Description	Gene markers	RUNX1		RUNX2		RUNX3	
		Cor	*P*	Cor	*P*	Cor	*P*
**CD8^+^T cell**	CD8A	0.24	***5.7e-05***	0.32	***8.3e-08***	0.48	***3.7e-17***
	CD8B	0.14	***0.02***	0.23	***0.00016***	0.33	***1.2e-08***
**T cell (general)**	CD3D	0.22	***0.00025***	0.31	***1.3e-07***	0.51	***5.5e-20***
	CD3E	0.33	***1.4e-08***	0.43	***1.1e-13***	0.59	***6.6e-27***
	CD2	0.32	***4.1e-08***	0.4	***8.7e-12***	0.57	***6e-25***
**B cell**	CD19	0.22	***0.00031***	0.31	***1.7e-07***	0.43	***4e-14***
	CD79A	0.31	***12e-07***	0.42	***6.8e-13***	0.54	***2e-22***
**Monocyte**	CD86	0.45	***6.9e-15***	0.58	***1.9e-26***	0.63	***4.6e-32***
	CD115 (CSF1R)	0.53	***1.5e-21***	0.64	***3.7e-33***	0.67	***1.7e-37***
**TAM**	CCL2	0.47	***9e-17***	0.57	***7.2e-25***	0.52	***7.5e-21***
	CD68	0.43	***5.3e-14***	0.52	***4.9e-20***	0.59	***3e-27***
	IL10	0.47	***2.5e-16***	0.54	***2.5e-22***	0.57	***2.2e-25***
**M1 Macrophage**	INOS (NOS2)	-0.14	***0.021***	-0.091	0.13	0.12	***0.094***
	IRF5	0.22	***0.00021***	0.24	***5.8e-05***	0.24	***5.8e-05***
	COX2 (PTGS2)	0.24	***6.3e-05***	0.33	***3e-08***	0.3	***3e-07***
**M2 Macrophage**	CD163	0.46	***8.1e-16***	0.64	***1e-33***	0.6	***1.4e-28***
	VSIG4	0.45	***3.2e-15***	0.6	***8.1e-28***	0.58	***1.7e-26***
	MS4A4A	0.45	***2.7e-15***	0.61	***1.5e-29***	0.61	***1.3e-29***
**Natural killer cell**	KIR2DL1	0.1	0.083	0.16	***0.0071***	0.22	***0.00021***
	KIR2DL3	0.16	***0.0062***	0.22	***0.00018***	0.25	***2.7e-05***
	KIR2DL4	0.054	0.37	0.15	***0.014***	0.28	***2.1e-06***
	KIR3DL1	0.095	0.12	0.25	***2.8e-05***	0.27	***5.5e-06***
	KIR3DL2	0.22	***2e-04***	0.33	***2.1e-08***	0.41	***8.8e-13***
	KIR3DL3	0.069	0.25	0.083	0.17	0.16	***0.0092***
**Dendritic cell**	HLA-DPB1	0.41	***1.6e-12***	0.53	***1.6e-21***	0.6	***1.6e-28***
	HLA-DQB1	0.22	***0.00032***	0.26	***9.1e-06***	0.29	***9.6e-07***
	HLA-DRA	0.32	***7.9e-08***	0.42	***6.2e-13***	0.53	***5.2e-21***
	HLA-DPA1	0.38	***1e-10***	0.47	***2.3e-16***	0.56	***1.4e-24***
	BDCA-1 (CD1C)	0.37	***1.4e-10***	0.42	***2.8e-13***	0.48	***2.4e-17***
	BDCA-4 (NRP1)	0.58	***4.2e-26***	0.69	***1.4e-39***	0.6	***3.4e-28***
	CD11c (ITGAX)	0.51	***1.5e-19***	0.66	***2.5e-35***	0.64	***1.3e-32***
**Th1**	T-bet (TBX21)	0.32	***5.8e-08***	0.4	***4e-12***	0.54	***2.1e-22***
	STAT4	0.38	***5.2e-11***	0.42	***2.1e-13***	0.53	***2e-21***
	STAT1	0.36	***6.7e-10***	0.35	***1.7e-09***	0.45	***6.5e-15***
	IFN-γ (IFNG)	0.15	***0.012***	0.19	***0.002***	0.39	***1.9e-11***
	TNF-α (TNF)	0.33	***2.2e-08***	0.39	***1.8e-11***	0.4	***8.6e-12***
**Th2**	GATA3	0.46	***5.4e-16***	0.52	***3.1e-20***	0.6	***1.1e-28***
	STAT6	0.26	***1.2e-05***	0.22	***0.00032***	0.31	***2.3e-07***
	STAT5A	0.44	***1.5e-14***	0.41	***1.3e-12***	0.43	***9.2e-14***
	IL13	0.26	***1.8e-05***	0.3	***5.9e-07***	0.34	***4.5e-09***
**Tfh**	BCL6	0.58	***5.8e-26***	0.57	***1.4e-25***	0.59	***4.4e-27***
	IL21	0.18	***0.0034***	0.21	***0.00057***	0.3	***3.5e-07***
**Th17**	STAT3	0.43	***5.7e-14***	0.37	***2.6e-10***	0.42	***2.1e-13***
	IL17A	-0.097	0.11	-0.16	***0.0092***	-0.051	0.4
**Treg**	FOXP3	0.43	***8.7e-14***	0.51	***3.2e-19***	0.59	***1e-26***
	CCR8	0.47	***1.3e-16***	0.51	***1.1e-19***	0.57	***1.2e-24***
	STAT5B	0.44	***1.5e-14***	0.41	***1.3e-14***	0.34	***1.1e-08***
	TGFβ (TGFB1)	0.52	***3e-20***	0.62	***2.4e-30***	0.61	***5.3e-29***
**Resident T cell**	ITGAE	-0.12	***0.042***	0.042	0.49	0.083	0.17
	CD69	0.38	***5.9e-11***	0.44	***1e-14***	0.55	***2e-23***
	CXCR6	0.27	***6e-06***	0.35	***1.5e-09***	0.52	***8.2e-21***
	NR4A1	0.1	0.098	0.13	***0.026***	0.091	0.13
	NR4A3	0.36	***7.4e-10***	0.47	***3.4e-16***	0.39	***1.7e-11***
**Cytotoxic T cell**	PRF1	0.3	***5.5e-07***	0.42	***6.2e-13***	0.53	***3.7e-21***
	IFNG	0.15	***0.012***	0.19	***0.002***	0.39	***1.9e-11***
	GNLY	0.1	***0.087***	0.23	***0.00014***	0.33	***1.3e-08***
	NKG7	0.22	***0.00029***	0.33	***2.6e-08***	0.47	***7.6e-17***
	GZMB	0.068	0.26	0.051	0.4	0.056	0.36
	GZMA	0.15	***0.013***	0.27	***7.3e-06***	0.41	***1e-12***
	CST7	0.38	***3.8e-11***	0.49	***9.1e-18***	0.59	***4.4e-27***
	TNFSF10	0.34	***9.4e-09***	0.26	***1.2e-05***	0.33	***1.4e-08***
**Exhausted T cell**	PD1 (PDCD1)	0.3	***4.5e-07***	0.36	***5e-10***	0.52	***2.1e-20***
	PDL1 (PDCD1LG2)	0.49	***2.6e-18***	0.6	***6.2e-28***	0.63	***8.5e-32***
	CTLA4	0.4	***6.6e-12***	0.44	***1.8e-14***	0.55	***1.6e-23***
	LAG3	0.23	***0.00014***	0.29	***1e-06***	0.45	***3.8e-15***
	TIM-3 (HAVCR2)	0.46	***1.8e-15***	0.59	***3.6e-27***	0.63	***1.7e-31***
	TIGIT	0.36	***4.7e-10***	0.43	***4.1e-14***	0.57	***1.2e-24***
**Effector memory T cell**	GZMK	0.35	***2.6e-09***	0.45	***4.8e-15***	0.57	***4.9e-25***
	CXCR4	0.47	***1.2e-16***	0.53	***1.2e-21***	0.51	***2.5e-19***
	CXCR3	0.19	***0.0015***	0.17	***0.0058***	0.29	***8.1e-07***
	CD44	0.27	***5.7e-06***	0.076	0.21	0.13	***0.035***

Bold italic signifies *P* < 0.05.

### Correlation between the expression levels of RUNX1, RUNX2, RUNX3 and tumor infiltrating CD8^+^T cells in colorectal cancer

**Figure 8:**
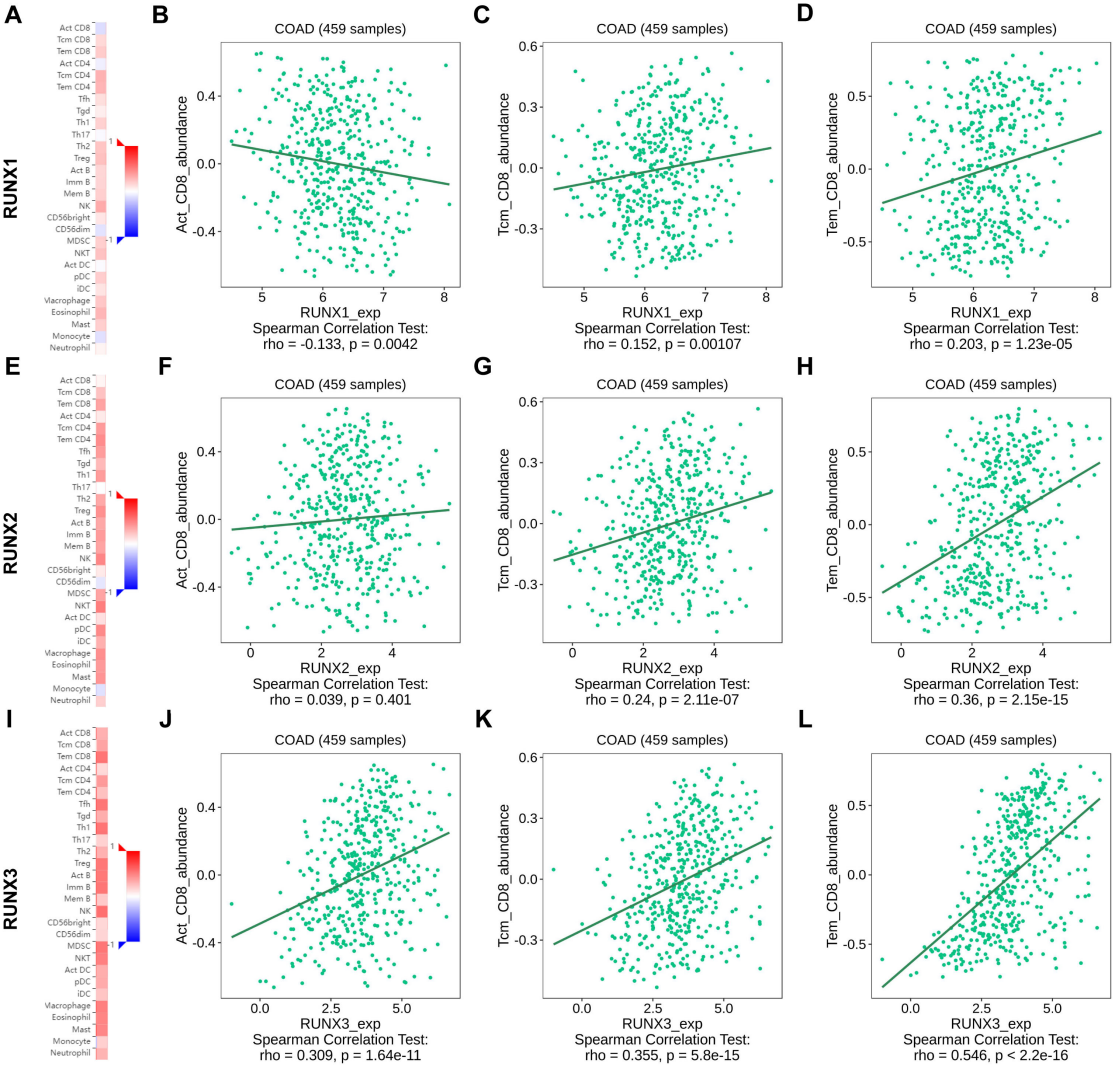
The association between RUNX family gene and immune infiltrating CD8^+^T cell subsets in human colorectal cancer based on TISIDB platform. **(A, E, I)** Heatmap showed the correlation of RUNX1 **(A)**, RUNX2 **(E)** and RUNX3 **(I)** expression and immune infiltration in pan-cancer. **(B–D)** Correlation between RUNX1 and active CD8^+^T, central memory CD8^+^T and effector memory CD8^+^T. **(F–H)** Correlation between RUNX1 and active CD8^+^T, central memory CD8^+^T and effector memory CD8^+^T. **(J–L)** Correlation between RUNX1 and active CD8^+^T, central memory CD8^+^T and effector memory CD8^+^T.

Upon closer examination of the relationship between the RUNX family gene and infiltrating CD8+T cells, it was observed that the values of Spearman’s correlation test rho exhibited a gradual increase from RUNX1 to RUNX3 on the TISIDB platform (**Figures 8A, E, I**). For instance, the spearman correlation rho was -0.133 between RUNX1 and active CD8^+^T (Act_CD8), data statistically significant, revealed a negative correlation between RUNX1 and active CD8^+^T cells (**Figure 8B**). While the rho value was 0.039 between RUNX2 and active CD8^+^T with a 0.401 P value, while revealed a none significant statistically correlation (**Figure 8F**). And the rho value became 0.309 between RUNX3 and active CD8^+^T, with statistically significant data again (**Figure 8J**). Similarly, the Spearman correlation coefficient rho between RUNX1, RUNX2 to RUNX3 and central memory CD8^+^T (Tcm_CD8) cells showed a stepwise increase (**Figures 8C, G, K**), so did the effector memory CD8^+^T (Tem_CD8) (**Figures 8D, H, L**). Besides, the Spearman’s correlation test rho value also presented that the correlation between RUNX genes with active CD8^+^T, central memory CD8^+^T and effector memory CD8^+^T were progressively increased, respectively (**Figures 8B–D, F–H, J–L**). These results indicated that RUNX family genes might be more relevant to effector CD8^+^ T than to central memory CD8^+^ T cells and activated CD8^+^ T cells and RUNX3 showed more relevant to effector CD8^+^ T than to central memory CD8^+^ T cells and activated CD8^+^ T cells than RUNX2 and RUNX1.

**Figure 9:**
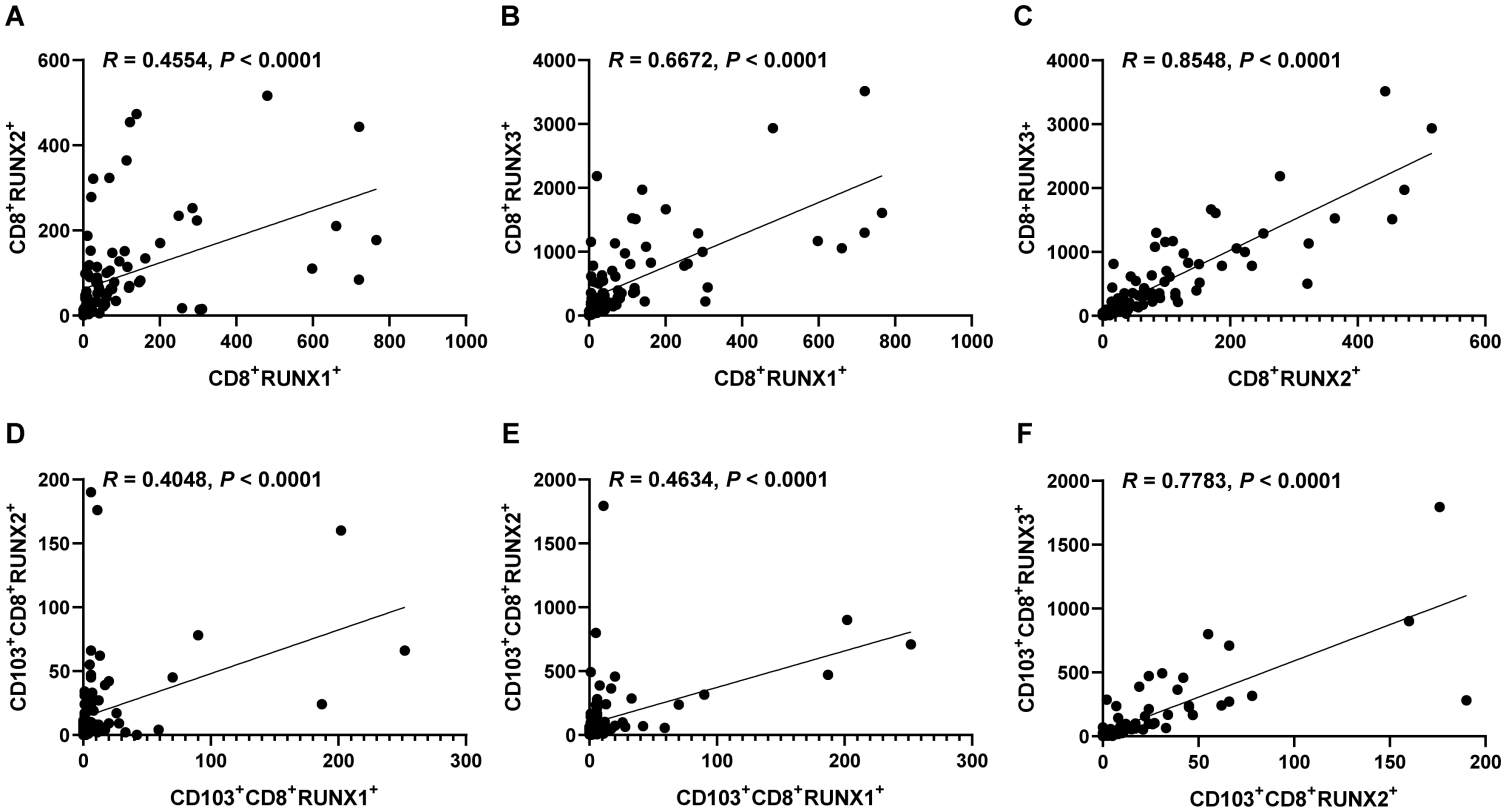
Correlations of the expressions of RUNX1, RUNX2 and RUNX3 in CD8^+^TILs and CD103^+^CD8^+^TILs in human CRC TMA. **(A)** The dot plot showed the correlation between CD8^+^RUNX2^+^T and CD8^+^RUNX1^+^T. **(B)** The dot plot showed the correlation between CD8^+^RUNX3^+^T and CD8^+^RUNX1^+^T. **(C)** The dot plot showed the correlation between CD8^+^RUNX3^+^T and CD8^+^RUNX2^+^T. **(D)** The dot plot showed the correlation between CD103^+^CD8^+^RUNX2^+^T and CD103^+^CD8^+^RUNX1^+^T. **(E)** The dot plot showed the correlation between CD103^+^CD8^+^RUNX3^+^T and CD103^+^CD8^+^RUNX1^+^T. **(F)** The dot plot showed the correlation between CD103^+^CD8^+^RUNX3^+^T and CD103^+^CD8^+^RUNX2^+^T.

**Figure 9** illustrates significant positive correlations between the expressions of RUNX1 and RUNX2, RUNX1 and RUNX3, as well as RUNX2 and RUNX3 in both CD8^+^T cells and CD103^+^CD8^+^T cells within the context of human CRC TMA in our mIHC findings. The correlation coefficients between CD8^+^RUNX2^+^T and CD8^+^RUNX3^+^T, as well as CD103^+^CD8^+^RUNX2^+^T and CD103^+^CD8^+^RUNX3^+^T, were notably higher compared to other comparison groups (R=0.8548, *P*<0.0001, **Figure 9C**, R=0.7783, *P*<0.0001, **Figure 9F**, respectively).

## Discussion

Recent studies have shown that each member of the RUNX family may play a role in various stages of tumor development, such as cell proliferation, apoptosis, and metastasis, indicating their potential as targets for diagnostic and therapeutic interventions in cancer. For instance, RUNX1, including its mutations, has been reported to serve as a prognostic factor in tumors including leukemia, myeloid neoplasia, and renal cancer (39–41). RUNX2 has been reported to be a novel prognostic signature and correlate with immune infiltrates in bladder cancer, cervical cancer and gastric cancer (25, 26, 42). RUNX3 has been found elevated in renal cancer and is associated with shorter progression-free survival, and high RUNX3 expression predicts a greater benefit of IO/TKI (immune checkpoint inhibitor combined with a lorosine kinase inhibitor) therapy compared to TKI monotherapy (43). In our current study, we initially examined the expression of all the three RUNX genes across different tumors using the TCGA database (**Figure 1**). By analyzing data from GEPIA, UALCAN, and TIMER, three widely utilized TCGA database platforms, we observed that the expression patterns of RUNX1 and RUNX2 were quite similar across most tumors, both showing increased expression levels, whereas RUNX3 was more frequently down-regulated in a larger number of tumors (**Figure 1J**). These findings align with previous reports on RUNX expression in pan-cancer contexts (44, 45), suggesting that abnormal RUNX expression is linked to prognosis in various cancers.

In the next section, we explored the relationship between RUNX family and prognosis. According to our mIHC results from **Figure 2**, especially **Figures 2D–F**, there was an up-regulation of RUNX1 expression and a down-regulation of RUNX3 expression in CRC TMA tumor tissues. The result was also consistent with our previous findings in the UALCAN database (**Figures 1A–C**). The different expression status of RUNX family members in the same one COAD tumor type, made us further explore their correlation with immune cells in the surrounding immune microenvironment. It has been shown that tumor-infiltrating lymphocytes (TILs) within the TME are a reliable determinant of prognosis and immunity to immunotherapy (46–48). Our study demonstrated that RUNX family gene were all strongly associated with TILs and played a key role in the development of TMEs (**Figure 7**, **Table 6**).

The RUNX family is essential for lineage characterization of various types of hematopoietic cells, including T lymphocytes (11, 12, 49). Current research has shown that the RUNX gene family plays a critical role in regulating the development, function, and immune response of CD8^+^T cells. RUNX1 and RUNX3 play important regulatory roles in promoting the development of immature CD4^+^CD8^+^ double-positive (DP) thymocytes into mature CD4^+^Th and CD8^+^CTL cells. RUNX1 binds to CD4 to silence and inhibit transcription in immature double-negative thymocytes while activating CD8 progression from double-negative to double-positive thymocytes. RUNX3, on the other hand can bind the core sequence of CD4 silencing and establish epigenetic silencing in CD4^-^CD8^+^ cytotoxic T cells (12). One of the major targets of tumor immunotherapy is to rescue and/or maintain optimal effector CD8^+^ T-cell function by minimizing tumor-induced negative factors. RUNX family proteins establish a core transcriptional program in CD8^+^CTL cells. T cells acquire specific effector functions by preparing chromatin landscapes early in development, a process that involves the sequential cooperation of transcription factors such as RUNX1, PU.1, and BCL11B, which sequentially and synergistically anchor mSWI/SNF with RUNX1 to balance the T cell effector landscape (50). RUNX3 acts as an upstream transcription factor that binds directly to cis-regulatory regions of Prf1 (which encodes perforin) and Eomes (a type of T-box protein) regulatory region, with T-box driving the gene expression program in activated CD8^+^CTL cells (13). RUNX3 is also recognized as a central regulator of tissue-resident memory CD8^+^T (T_RM_) cell development (51). The integrin CD103 on T_RM_ cells is a target gene of RUNX3. In CD8^+^ T cells stimulated by antigen receptors, RUNX3 is essential for establishing the signature cytotoxic effector function of CTLs to promote their clearance from tumors in the microenvironment. In addition to RUNX1 and RUNX3, RUNX2 also affects the development of memory CD8^+^T cells (52). In a murine model of acute lymphocytic choroidal meningitis virus infection, RUNX2 has been identified as a crucial factor for the maintenance of long-term memory CD8^+^ T-cell persistence and for influencing the development of memory CD8^+^ T cells (52). In our previous studies, we found that tumor infiltration CD8^+^T and CD103^+^CD8^+^T could serve as good clinical prognostic indicators in CRC (38). We also confirmed this conclusion at the beginning of this study (**Figure 3**). So, in our present study, we further analyzed the prognostic values of RUNX1, RUNX2 and RUNX3 expressed on CD8^+^TILs and CD103^+^CD8^+^TILs in human CRC. According to the results from **Figures 4**–**6**, **Table 5** in this study, we found that CD8^+^RUNX1^+^T, CD8^+^RUNX2^+^T, CD8^+^RUNX3^+^T and CD103^+^CD8^+^RUNX3^+^T cells could be important prognostic predictors for the survival prediction of CRC patients. Some previous researches have mentioned that CROX (Cluster Regulation of RUNX) could serve as a potential novel therapeutic approach for tumors (53, 54). In this study, our analysis of correlation between the expression levels of RUNX1, RUNX2 and RUNX3 in infiltrating CD8^+^T and CD103^+^CD8^+^T cells in colorectal cancer, implicated that the transcription factors RUNX1, RUNX2, and RUNX3 might exhibit a synergistic interaction effect within clusters and mutually influence each other (**Figure 9**). Although the comprehensive RUNX family cluster regulation has yet to be applied in the foundational and clinical fields, manipulating the RUNX gene family may have potential applications in immunotherapy, further study in RUNX gene family cluster will provide new insights and strategies for tumor immunotherapy.

The following defects and limitations may exist in this paper: 1) Since we used commercial tissue microarrays, insufficient sample size and insufficient sample resource may be limited. In subsequent research, more intraoperative clinical fresh tissue samples, in addition to the patient’s tumor and adjacent normal tissue, peripheral blood samples could also be included, to further validate the exploratory conclusions drawn in this paper. 2) This article does not provide a detailed study on how the RUNX family members, including RUNX1, RUNX2, and RUNX3, specifically regulate the mechanisms of tumor-infiltrating CD8^+^ T cells and CD103^+^CD8^+^ T cells in colorectal cancer, as well as the functional roles they play in the anti-tumor immune response within the CRC microenvironment. It has been reported that in antigen-stimulated CD8^+^ T cells, RUNX is crucial for developing CTL’s cytotoxic function and enhancing tumor clearance in the TME (13). The RUNX family members also appear to play a critical role in regulating the effector differentiation of effector T cells (T_eff_) and memory T cells (T_mem_) *in vivo*. For instance, within an *in vivo* T-cell CRISPR screening platform, the ETS family transcription factor Fli1 was identified as a repressor of T_eff_ cell biological function. The deletion of Fli1 led to increased chromatin accessibility at RUNX motifs, thereby enhancing the biological efficacy of RUNX-driven Teff cells, which demonstrated increased resistance to infection and tumorigenesis (55). Furthermore, existing literature indicates that the reversal of RUNX methylation facilitates CD8^+^ TILs infiltration, mitigates CD8^+^ T cell exhaustion, and augments the anti-tumor immune response of CD8^+^ T cells (56). Studies in pancreatic ductal adenocarcinoma and hepatocellular carcinoma have found that RUNX gene-edited CAR-T cells showed enhanced persistence, cytotoxic potential and tumor-resident capacity, and could enhance anti-tumor effects (57, 58). These studies indicate that the RUNX family may play a significant role in the functional differentiation of T effector cells, thereby establishing a theoretical foundation for considering RUNX as a potential target for predicting response rates to immunotherapy. The mechanism through which the RUNX family augments anti-tumor efficacy in CRC by modulating the effector functions of tumor-infiltrating CD8^+^ T cells and CD103^+^CD8^+^ T cells deserve further elucidation.

To sum up, our recent research highlights the prognostic importance of the infiltration intensity of RUNX family genes in CD8^+^T and resident CD103^+^CD8^+^T cells in human colorectal cancer. The RUNX family could be a vital and promising prognostic biomarker for forecasting disease progression and immune evasion in colorectal cancer patients.

## Data Availability

The datasets presented in this study can be found in online repositories. The names of the repository/repositories and accession number(s) can be found in the article/supplementary material.

## Glossary

RUNX: runt-related transcription factor
CRC: colorectal cancer
TME: tumor microenvironment
PD-1: programmed death 1
PD-L1: programmed cell death 1 ligand 1
CAR-T: chimeric antigen receptor T-cell immunotherapy
TIL: tumor-infiltrating lymphocyte
CXCL13: chemokine C-X-C motif ligand 13
TGF-β: transforming growth factor beta
BMP: bone morphogenetic protein
YAP1: Yes-associated protein 1
mIHC: multi-color immunohistochemically staining
DAPI: Diamidino-2-phenylindole
DC: dendritic cell
TAM: tumor-associated macrophage
TCGA: The Cancer Genome Atlas
BLCA: bladder urothelial carcinoma
BRCA: breast carcinoma
CESC: cervical squamous cell carcinoma and endocervical adenocarcinoma
COAD: colon adenocarcinoma
DLBC: lymphoid neoplasm diffuse large B-cell lymphoma
ESCA: esophageal carcinoma
GBM: glioblastoma multiforme
HNSC: head and neck squamous cell carcinoma
KICH: kidney chromophobe
KIRC: kidney renal clear cell carcinoma
KIRP: kidney renal papillary cell carcinoma
LAML: acute myeloid leukemia
LGG: lower-grade glioma
LIHC: liver hepatocellular carcinoma
LUAD: lung adenocarcinoma
LUSC: lung squamous cell carcinoma
PAAD: pancreatic adenocarcinoma
PRAD: prostate adenocarcinoma
SKCM: skin cutaneous melanoma
STAD: stomach adenocarcinoma
TGCT: testicular germ cell tumors
THCA: thyroid carcinoma
THYM: thymoma
UCEC: uterine corpus endometrial carcinoma
UVM: uveal melanoma
